# The Capacity of Periodontal Gel to Occupy the Spaces Inside the Periodontal Pockets Using Computational Fluid Dynamic

**DOI:** 10.3390/dj8010001

**Published:** 2019-12-24

**Authors:** Luca Levrini, Luigi Paracchini, Maria Giulia Nosotti

**Affiliations:** 1 Department of Human Sciences, Innovation and Territory, Dental Hygiene School, University of Insubria, 22100 Varese-Como, Italy; luca.levrini@uninsubria.it; 2 INGEO, 28100 Varallo Pombia (Novara), Italy; luigi.paracchini@ingeosnc.it

**Keywords:** Computional Fluid Dynamic, CAT scan, periodontal gels

## Abstract

The aim of the current work is to demonstrate the capacity of a new periodontal gel to occupy the spaces inside the periodontal pockets through Computational Fluid Dynamic (CFD). The test gel consists of two resorbable medical grade polymers (PEO, Poly Ethylen Oxide and HPMC, Hydroxy Propyl Metyl Cellulose), Type I Collagen, SAP (Vitamin C), and PBS (Saline Solution), while the control gel is 14% doxyclin controlled release gel, which is used for treating periodontal pockets with probing ≥5 mm after scaling and root plaining. The study examined the fluid dynamic analysis (Computational Fluid Dynamic—CFD) of two different gels, used in dentistry to treat periodontitis, in relation to both the geometry of the periodontal pocket and the function of two different types of needles that are used to distribute the preparation. The periodontal pocket was determined by reading DICOM images taken from the patient’s CAT scan. The results show that the H42^®^ gel comes out uniformly compared to the other gel. Moreover, it is possible to observe how the rheological properties of the gel allow the fluid to spread evenly within the periodontal pocket in relation to the geometry of the needle. In particular, H42^®^ gel exits in a constant way both from the first and the second exit. In fact, it was observed that by changing the geometry of the needle or the type of periodontal gel, the distribution of the gel inside the pocket was no longer homogeneous. Thus, having the correct rheological properties and correct needle geometries both speeds up the gel and optimizes the pressure distribution. Currently, the literature is still lacking, therefore further studies will be needed to confirm these results.

## 1. Introduction

Fluids are substances of which their molecular structure offers no resistance to external forces—in fact, even the smallest force can deform a fluid particle [1].

Computational Fluid Dynamics (CFD) provides a qualitative prediction of fluid flows by means of [2]:

- Mathematical modeling (partial differential equations)
- Numerical methods (discretization and solution techniques)
- Software tools (solvers, pre- and post-processing utilities)

CFD allows us to carry out “numerical experiments” (i.e., computer simulations) in a “virtual flow laboratory”.

Fluid flow is caused by the action of externally applied forces, such as differences in pressure, gravity, shear, rotation, and surface tension [2].

While all fluids behave similarly under the action of forces, their macroscopic properties differ considerably. We must be aware of the most important properties of simple fluids, which are density and viscosity [1].

The aim of the current work was to demonstrate the capacity of a new periodontal gel to occupy the spaces inside the periodontal pockets through Computational Fluid Dynamic (CFD).

The most common periodontal gels that are reported in the literature are:

- Tetracycline: a broad-spectrum naphthacene antibiotic, which binds to the 30 S ribosomal subunit, inhibiting protein synthesis [3]. In dentistry, it can be found in the form of tetracycline fiber (Periodontal Plus AB). This product is effective, easy to place, and has few side effects and adverse consequences. However, further long-term studies are needed to evaluate the regular use of this product [4].
- Minocycline: a derivative of tetracycline with excellent absorption and tissue penetration that is used for several bacterial infections. It is a slow release product of good consistency and with antibacterial activity [5]. In dentistry, it can be found in the form of microspheres (Arestin) and ointment (Periocline). According to Matesanz-Pérez et al., the different studies that were used in their meta-analysis suggested a positive effect of minocycline on the reduction of probing depth, but not on the onset [6].
- Metronidazole: derived from nitronidazole and has antibacterial activities. Non-ionized metronidazole is absorbed by obligate anaerobic organisms and is then reduced by transport proteins into an active intermediate product. Thus, it inhibits DNA synthesis and bacterial cell growth [7]. Elyzol can also be found in dentistry and it is a gel that is composed of a solid suspension of metronidazole benzoate with a moniglyceride and a triglyceride.
- Doxycycline: a broad-spectrum antibiotic with antimicrobial activity. It binds to the 30 S ribosomal subunit and blocks the binding of aminoacyl-tRNA to the mRNA-ribosome complex, thereby inhibiting protein synthesis. In addition, this agent has exhibited inhibition of collagenase activity. Ligosan can be found in dentistry and it appears to have two powerful effects: (1) if administered with a systemic antibiotic, it increases its effectiveness; (2) maximum effectiveness occurs when the periodontal pocket is treated in the active phase with bleeding in the probing and/or pus [8]. Ligosan complements the standard non-surgical procedure (SRP) for periodontal pocket treatment. The product remains in the correct position and after 12 days it undergoes a spontaneous process of biodegradation and bio-absorption which does not require removal [9].
- Chlorhexidine Gluconate: an antiseptic agent with topical antibacterial activity. It is positively charged and reacts with the negatively charged microbial cell, thus destroying the cell membrane. For this reason, Gram-positive bacteria, which have a more negative charge, are more sensitive to this product [10]. Periochip, which is used in dentistry, has been found to reduce pocket depth after causal therapy [11].
- There are viscous solutions of sulfates that are added to sulfuric acid which make use of the drying properties of the polysaccharide matrix of the biofilm cells. By absorbing the water from the biofilm, the bacteria, and their matrix, this causes a clot and therefore, the collapse of the internal structure of the plaque [12]. HybenX can be found in dentistry and the liquid is placed in the pocket, kept for a minute, and then rinsed. Tooth sensitivity during application has occurred [12].

In the literature, it is clear that the active ingredients of the aforementioned gels (antibiotics and chlorhexidine) can cause some unwanted side effects: the appearance of allergies, the appearance of candidiasis, and bacterial resistance [13,14,15].

Given this vast pharmacological offer, we have analyzed the penetration capacity of a new innovative product in the periodontal pocket. We believe that it is important that the product, regardless of its effectiveness, reaches the periodontal pocket in depth. The factors that determine this capacity are the viscosity of the product and the inoculation mode [16,17]. The aim of this study was to compare the effectiveness of two periodontal gels for the treatment of periodontal pockets using Computational Fluid Dynamic (CFD).

The product that was analyzed for testing is called Hydrogel H42^®^ (Arxé Sagl, Paradiso, Switzerland). It is a Class III Medical Device that is used for filling, reinforcement, and repair of periodontal/peri-implant pockets due to periodontitis and peri-implantitis. Thanks to its rheological, chemical-physical, and biochemical properties, Hydrogel H42^®^ can be used as adjuvant gel in the non-surgical treatment of periodontal pockets and peri-implantitis after the mechanical removal of plaque (scaling) and root planning of the pocket.

Hydrogel 42^®^ is based on a proprietary, polymeric hydrogel (Exur^®^, Bioteck Spa, Arcugnano, Italy).

## 2. Materials and Methods

The study considered the fluid dynamic analysis (Computational Fluid Dynamic—CFD) of two different gels, used in dentistry to treat periodontitis, in relation to both the geometry of the periodontal pocket and the function of two different types of needles that are used to distribute the preparation.

The gel contains:

1. Poly Ethylene Oxide (PEO): a resorbable medical polymer with high molecular weight. It provides rheological properties, longer degradation times, and physical stability to the gel [18].
2. Hydroxypropyl Methylcellulose (HPMC): is a resorbable medical polymer derived from cellulose. It provides rheological properties and high hydrophilicity for the gel [19].
3. Type I Collagen: of equine origin, needed for the consistency of the product, and has hydrophilic and haemostatic characteristics [20].
4. Vitamin C: is an antioxidant agent that is synthesized by plants and most animals [21]. According to the study of Sànchez-Najera, Vitamin C has important bactericidal activity against Streptococcus mutans, Staphylococcus aureus, Porphyromonas gingivalis, Candida albicans, and Enterococcus faecalis [22]. The action of Vitamin C on the polymerization of the polymers maintains the rheological properties both before and after sterilization, ensuring fluidity of the gel and prolonged times of reabsorption (at least 14 days). It presents: visco-elasticity, mucus-adhesiveness (mucosal adhesiveness, gums), physical stability, and the ability to remain stable in a liquid environment (saliva, blood).

Thanks to these components, the gel is viscous and remains inside the working site for several days. The control gel is 14% doxyclin controlled release gel, which is used for treating periodontal pockets with probing >5 mm after scaling and root plaining. The release of doxycycline in the gingival crevicular fluid is equal to 16 µg/mL for 12 days. Its matrix consists of polyglycolides and macrogols, which allow both the regulation of the increase in viscosity and the controlled release of the active principle in situ. The product is not eliminated after the treatment because the excipients are biodegradable in ethylene glycol, lactic acid, and glycolic.

With reference to the different working conditions outlined above, the periodontal pocket was determined in relation to DICOM images taken from the patient’s CAT scan.

**Figure 1:**
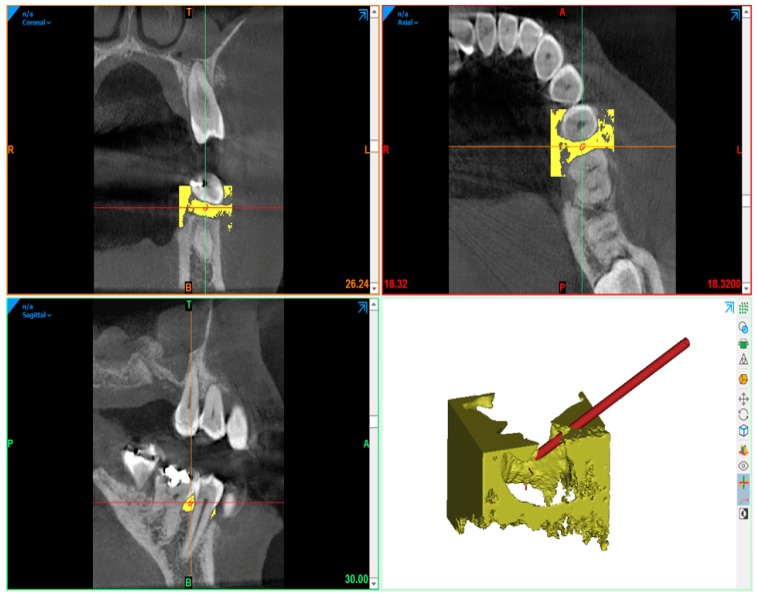
Images extracted from DICOM files obtained by CAT scan from the patient analysis and three-dimensional reconstruction of the pocket. In particular, it is possible to observe the positioning of the needle that will be used to make the gel penetrate inside the pocket.

The earliest signs of periodontitis can be detected by X-ray, which generate a wedge-shape radiolucent area in the inter-proximal region [23,24]. During CAT reconstruction, the absorption coefficient of radiation was used within a tissue to produce a grayscale image, called the Hounsfield scale, which is a quantitative measure of density. The Hounsflield unit was calculated on the basis of a linear transformation of the basic linear attenuation coefficient of the x-ray beam, where a dense tissue, with greater X-ray absorption, has positive values and is brighter, whereas the less dense tissue, with less X-ray absorption, has negative values and is darker [25]. In Figure 1, it is possible to observe one of the DICOM slices, taken from random among the other images, which shows the three-dimensional reconstruction area of the periodontal pocket. Obtaining the patient’s CAT scan, the extraction of the individual images, and the three-dimensional reconstruction of the patient’s periodontal pocket was carried out using RHINOCEROS 6.0 SR10 (6.10.18242.16.581) software in WINDOWS 10_x64 2016 ENTERPRISE LTSB environment with HP hardware (workstation Z240).

**Figure 2:**
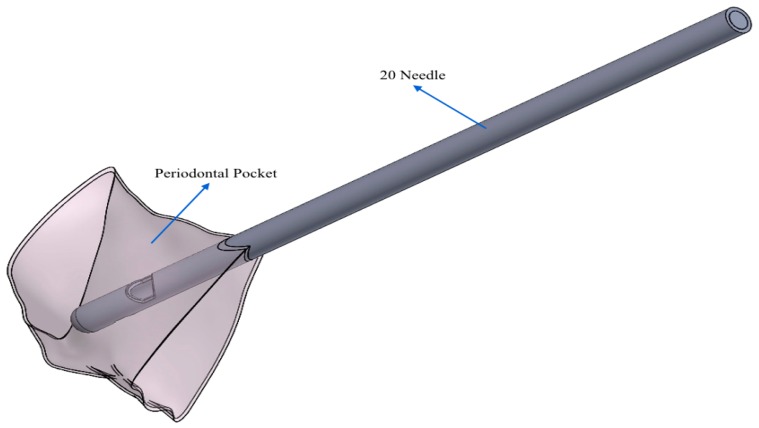
In this case, the periodontal pocket was placed in contact with the 20 G needle. The vision of the periodontal pocket was set in transparency to better show the interior between the pocket itself and the 20 G needle.

**Figure 3:**
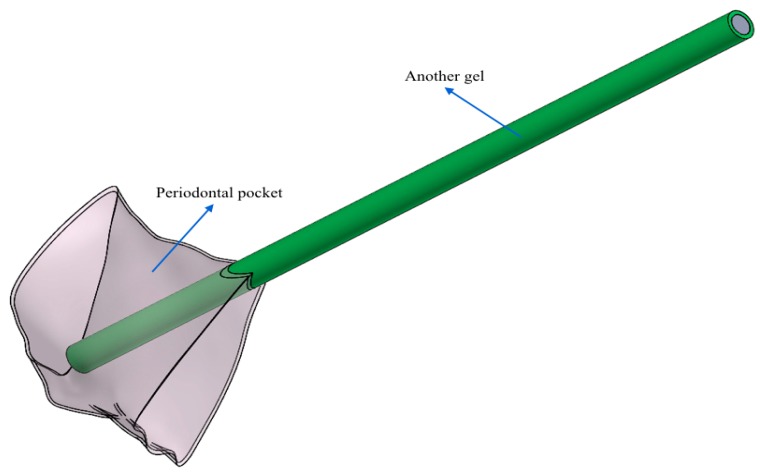
In this case, the periodontal pocket was placed in contact with another needle. The vision of the periodontal pocket was set in transparency to better show the interior between the pocket itself and another needle.

In particular, Figure 2 and Figure 3 show the finished periodontal pocket, geometrically optimized and placed in relation to the two different types of needles that were used to introduce the gel.

**Figure 4:**
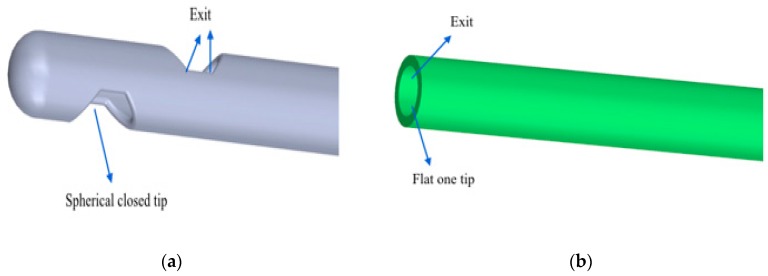
(**a**) 20 G needle that was used to spread the gel inside the periodontal pocket. It is possible to observe a closed spherical tip and two exits; (**b**) another needle that was used to spread the gel inside the periodontal pocket. It is possible to observe a flat open tip and an exit.

On the basis of the geometric information shown in Figure 2 and Figure 3, in Figure 4a,b, it is possible to observe the different geometric shape of the needles.

As with the three-dimensional model of the pocket, the SOLIDWORKS 2014 SP4 software was also used in the WINDOWS 10_x64 2016 ENTERPRISE LTSB environment to model the two needles. To complete the geometric information of the two needles, a length of 20 cm was considered (this length is the path that the gel travels from the containment tank up to its exit and was one of the parameters that was used for the CFD simulation). Below are the parameters that were used for the CFD of H42^®^ gel and of the other gel (Table 1):

In detail, below (Table 2), the characteristic rheological data relating to the two gels are presented.

All CFD analyses were conducted considering an internal simulation condition, excluding any cavities without a flow condition, in a laminar, and turbulent flow regime at a pressure of 101,325 Pa at a body temperature of 37 °C (330 K).

**Figure 5:**
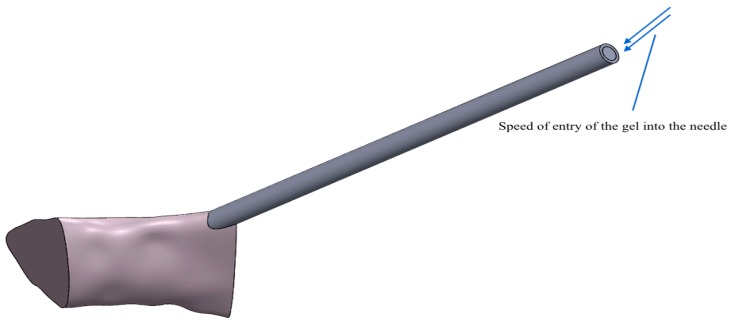
Indication regarding the entry speed (4 m/s) of the gel from the tank towards the needle.

Finally, we hypothesized a speed of entry of the gel inside the needle of about 4 m/s. This speed is presumably the speed at which the gel is fired, by means of a pressure system, from the tank versus the needle (Figure 5).

Therefore, in the pocket, we are at ambient pressure or 101,325 Pa. By injecting the H42^®^ gel, we obtain, at the exit of the syringe, a pressure of 101,857 Pa and therefore, the difference is 532 Pa. In contrast, by injecting the other gel, a pressure of 102,054 Pa is obtained for the entire output of the syringe and therefore, the difference is 729 Pa. The pressure in the pocket increases by about 35% if the other gel is injected. This aspect is detrimental to the strength of the walls of the pocket.

Moreover, by means of the DICOM technique, it was possible to compare the two different types of needles that were used to introduce the gel. The two compared needles had the following characteristics:

1. 20 G needle (test gel): closed spherical tip and two exits;
2. Another needle (control gel): flat open tip and one exit.

## 3. Results

Computational fluid dynamics (CFD) studies the mechanics of fluids using algorithms, which in addition to being used in engineering and in industry, is also used in medicine [26].

**Figure 6:**
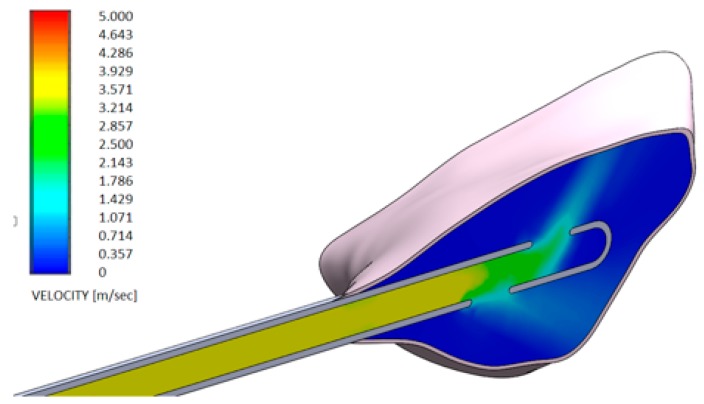
Velocity profile of the H42 GEL using a 20 G needle (spherical closed-ended needle and two openings—see also Figure 4a).

**Figure 7:**
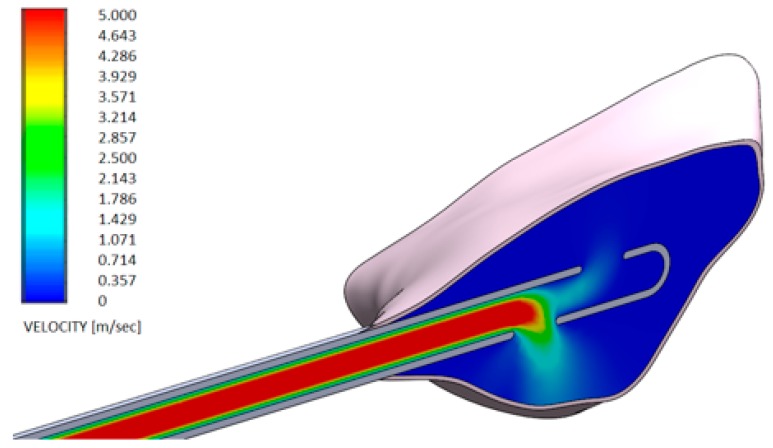
Velocity profile of another GEL using a 20 G needle (spherical closed-ended needle and two openings—see also Figure 4a).

**Figure 8:**
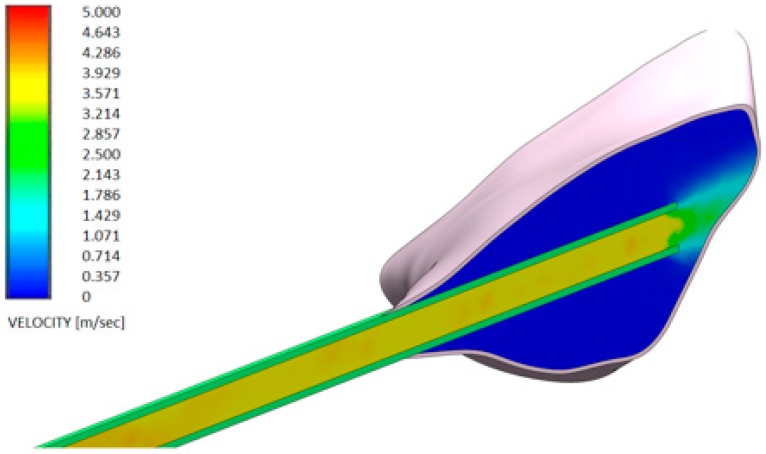
Velocity profile of the H42 GEL using a flat point needle (flat open-tipped needle—see also Figure 4b).

**Figure 9:**
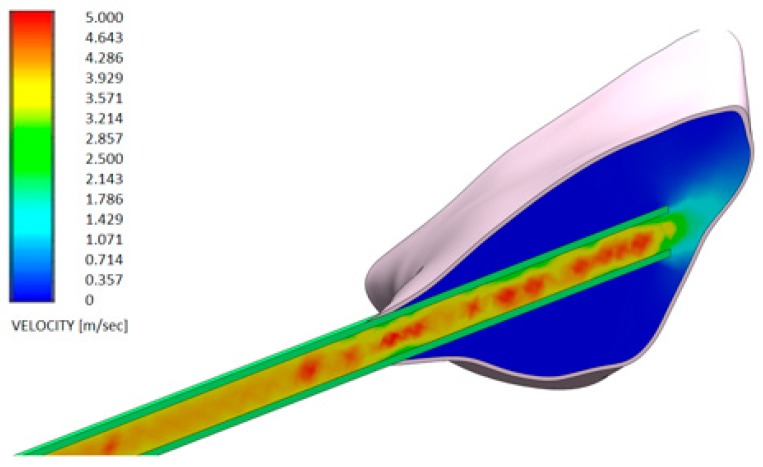
Velocity profile of another GEL using a flat point needle (flat open-tipped needle—see also Figure 4b).

Figure 6 and Figure 7 show the distributions of the outlet velocity of the gel from the needle inside the periodontal pocket, which are obtained by means of a central cutting plane. Figure 8 and Figure 9 highlight the velocity profile.

The periodontal pocket was determined using DICOM images taken from the patient’s CAT scan. The CFD analysis was conducted using FLOW SIMULATION 2014 SP4 (4123) in a WINDOWS 10_x64 2016 ENTERPRISE LTSB environment. DICOM (Digital Imaging and Communication in Medicine) is used in medicine to transmit radiological and other medical information between computers and various devices [27].

The output speeds of the gels from the needle inside the periodontal pocket were compared:

1. Test gel + 20 G needle: the gel exits uniformly without speed peaks, both from the first and second exit, thanks to the rheological properties of the gel and the geometry of the needle;
2. Control gel + 20 G needle: the liquid penetrates inside the pocket only from the first exit;
3. Test gel + Flat Point needle and Another Gel + Flat Point needle: the gel remains confined to the tip, so it has an uneven distribution within the periodontal pocket.

It is also important to consider pressure. In fact, the correct combination of rheological characteristics of the gel and the geometric shape of the needle optimize both the exit speed of the gel and the pressure distribution.

Therefore, changing the type of gel and the geometry of the needle would mean totally modifying the results obtained by CFD in regards to the distribution of speed and pressure.

## 4. Discussion

Thanks to the use of CFD, it was possible to simulate the output speed of the gels in the pocket. As can be seen from the results obtained, if the H42^®^ gel is injected with a 20 G needle, the gel exits uniformly without speed peaks. By contrast, if you use another gel with a 20 G needle, the gel enters the pocket only from the first exit, while if H42^®^ gel with another needle is used, the gel remains confined to the tip, so the distribution is irregular. Periodontal diseases are dangerous for the periodontium due to the bacterial etiology. This bacteria can spread to systemic circulation. The destruction of tissue is due to the overproduction of free radicals and reactive oxygen species and to the matrix metalloproteinases, which causes the breakdown of collagen and periodontal cells [28].

Other important factors for the destruction of the periodontium are oxidative stress and diminished antioxidant capacity [29,30].

In daily practice, for the treatments of periodontal disease, periodontal gels can be used. The most frequently used gels for the treatment of periodontal disease are gels with antibiotics, such as: Tetracycline, Minocycline, Metronidazole, and Doxicicline. These gels are more effective for local than systemic use, even though periodontal pathogens are more sensitive in vivo than in periodontal pockets, because the gel tends not to stay in the pockets. Other types of gels are Chlorhexidine gluconate and Sulfate Solution, however even these gels tend not to stay in situ.

The side effects of these gels can be the appearance of allergies, the appearance of candidiasis, and bacterial resistance.

The gels that were compared in this study are two hydrogels. Peptide-based hydrogels are an important class of biomaterials with various possible applications, including dentistry. To understand whether these biomaterials are suitable for biotechnological uses, it is important to understand the mechanical properties of the hydrogel and of the formation and deformation mechanisms. In the study of Yan et al., the rheological characteristics of the physical properties of the hydrogel based on peptides and polypeptides and the behavior of the hydrogel during and after the flow were evaluated [31]. A hydrogel is considered good if it has injectable methods of administration and if applications are reduced. Solid gels are able to remain at the site by simple syringe injection [31]. The importance of the correct combination of rheological characteristics of the gel and the geometric shape of the needle must be emphasized so as to have a correct exit speed of the gel and a correct pressure distribution. It is obvious that by changing the type of gel and the geometry of the needle, speed and pressure will be modified.

## 5. Conclusions

This study made it possible to compare various commonly used gels. In particular, the comparison took place between Hydrogel H42^®^ and another gel containing Doxycycline through fluid dynamics analysis. Thanks to the results that were obtained by inoculation of the two gels inside a periodontal pocket, it is possible to state that both the rheological properties of the gel and the geometry of the needle are fundamental factors for inserting and maintaining the periodontal gel in situ. In fact, it was observed that by changing the geometry of the needle or the type of periodontal gel, the distribution of the gel inside the pocket was no longer homogeneous. Thus, by having correct rheological properties and correct needle geometries, you optimize both the gel output speed and the pressure distribution. The literature is currently still poor, therefore further studies will be useful to optimize these very pioneering initial results.

## Figures and Tables

**Table 1:** Parameters used for the CFD of H42^®^ gel and of the other gel.

Global Mesh Settings
Automatic initial mesh: On
Result resolution level: 4
Advanced narrow channel refinement: On
Refinement in solid region: Off
Number of elements: 46,150 (elements used for two configurations)
Geometry Resolution
Evaluation of the minimum gap size: Manual
Minimum gap size: 5.000 × 10^−4^ m
Evaluation of the minimum wall thickness: Automatic
Initial Conditions
Thermodynamic parameters
Static Pressure: 101,325.00 Pa
Temperature: 293.20 K
Velocity parameters
Velocity vector
Velocity in X direction: 0 m/s
Velocity in Y direction: 0 m/s
Velocity in Z direction: 0 m/s
Material Settings
Fluids
GEL H42^®^–Other GEL
Boundary Conditions
Environment Pressure 1
Type: Environment Pressure
Faces: Real pocket-1/Cavity1//Face
Coordinate system: Global coordinate system
Reference axis: X
Thermodynamic parameters
Environment pressure: 101,325.00 Pa
Temperature type: Temperature of the initial components
Temperature: 293.20 K
Inlet Velocity 1
Type: Inlet Velocity
Faces: LID1-1/Imported1//Face
Coordinate system: Face Coordinate System
Reference axis: X
Flow parameters
Flow vectors direction: Normal to face
Velocity normal to face: 4.000 m/s
Thermodynamic parameters
Temperature type: Temperature of the initial components
Temperature: 293.20 K
Boundary layer parameters
Boundary layer type: Turbulent
Computational Domain
Size
X min: 0.026 m
X max: 0.045 m
Y min: 0.010 m
Y max: 0.030 m
Z min: 0.015 m
Z max: 0.035 m
X size: 0.019 m
Y size: 0.020 m
Z size: 0.020 m
Boundary Conditions
2D plane flow: None
At X min: Default
At X max: Default
At Y min: Default
At Y max: Default
At Z min: Default
At Z max: Default
Physical Features
Heat conduction in solids: Off
Time dependent: Off
Gravitational effects: Off
Rotation: Off
Flow type: Laminar only
High Mach number flow: Off
Free surface: Off
Default roughness: 0 micrometer

**Table 2:** Characteristic rheological data relating to the two gels.

Gel H42	Another Gel
Density 1.02 g/cm^3^; Specific heat 4182 J/(kg × K) Thermal conductivity 0.6 W (m × K) Viscosity POWER-LAW model 0.7991 Consistency coefficient 0.012171 Pa × s Maximum dynamic viscosity 0.012171 Pa × s Minimum dynamic viscosity 0.003038269 Pa × s	Density 0.917 g/cm^3^; Specific heat 4971 J/(kg × K) Thermal conductivity 0.39 W (m × K) Viscosity POWER-LAW model 1 Consistency coefficient 0.023765 Pa × s Maximum dynamic viscosity 0.023765 Pa × s Minimum dynamic viscosity 0.005405723 Pa × s
